# Socioeconomic Status and Depression Among Older Adults with Disabilities in Korea: The Mediating Role of Social Support and the Moderating Effects of Self-Esteem and Public Service Utilization

**DOI:** 10.3390/healthcare14101349

**Published:** 2026-05-14

**Authors:** Sanghyun Park, Joonhee Ahn

**Affiliations:** Department of Social Welfare for the Elderly, Hoseo University, Cheonan 31066, Republic of Korea; seasanghyun@naver.com

**Keywords:** socioeconomic status, depression, social support, moderated mediation, reserve capacity model, older adults with disabilities, public service utilization, health inequality

## Abstract

**Background/Objectives:** Depression among older adults with disabilities represents a significant public health concern, with well-documented socioeconomic disparities. However, the mechanisms through which socioeconomic status (SES) is associated with depression in this population remain insufficiently specified. Guided by the Reserve Capacity Model (RCM), this study examines whether social support is statistically associated with the SES–depression relationship and whether this association varies according to psychosocial and structural resources. **Methods:** Data were drawn from the 18th wave (2023) of the Korea Welfare Panel Study (KoWePS) and its supplementary disability survey (N = 845). Moderated mediation analyses were conducted using the PROCESS macro with 5000 bootstrap samples to estimate indirect and conditional associations. **Results:** SES was negatively associated with depressive symptoms and positively associated with social support. Social support demonstrated a statistically significant indirect association between SES and depression, consistent with a partial mediation pattern, although the direct association remained significant. Self-esteem did not significantly moderate the indirect association. In contrast, public service utilization significantly moderated the association between social support and depression, such that the indirect association between SES and depression was attenuated at higher levels of service utilization. **Conclusions:** The findings indicate that depression among older adults with disabilities is associated with both socioeconomic disadvantage and variations in social and structural resources. These results underscore the relevance of considering both psychosocial and structural dimensions of resources when examining mental health disparities.

## 1. Introduction

South Korea has rapidly transitioned into a super-aged society, becoming one of the fastest-aging countries in the world. While population aging is a global phenomenon, its pace and intensity in South Korea are particularly pronounced. Within this broader demographic shift, the aging of persons with disabilities is progressing at an even faster rate. According to the 2023 National Survey on Persons with Disabilities, individuals aged 65 and older account for 54.3% of all registered persons with disabilities [1], and prior research indicates that their rate of aging substantially exceeds that of the non-disabled population [2].

This demographic transformation reflects a broader global trend in which aging and disability increasingly intersect. However, institutional responses remain fragmented across aging and disability service systems, resulting in discontinuities in care and gaps in service provision. Consequently, older adults with disabilities represent a rapidly expanding population facing compounded vulnerabilities, including socioeconomic disadvantage, social isolation, and elevated health risks. Understanding the mechanisms that shape their health outcomes is therefore a critical public health priority, particularly for informing targeted interventions aimed at reducing health disparities in aging societies.

Among these health outcomes, depression is one of the most prevalent and consequential conditions. A substantial body of research has consistently demonstrated that individuals with disabilities experience higher levels of depression compared to the general population, with older adults with disabilities being especially vulnerable [3,4,5,6]. Depression in later life is associated with increased morbidity, diminished quality of life, and elevated suicide risk and is also linked to the progression of chronic diseases and cognitive decline [7,8,9,10,11,12].

A large and well-established body of empirical research has documented that lower socioeconomic status (SES) is associated with higher levels of depression, particularly in later life. Studies based on nationally representative samples have consistently shown that older adults with lower income, lower educational attainment, and disadvantaged living conditions are at greater risk of depressive symptoms [13,14,15,16]. Furthermore, this association tends to be more pronounced among individuals with disabilities, who often face compounded disadvantages such as functional limitations, reduced labor market participation, and restricted access to social resources [17,18]. Recent studies focusing specifically on older adults with disabilities have similarly reported that socioeconomic disadvantage is strongly associated with poorer mental health outcomes, including higher levels of depression and psychological distress [19,20,21,22].

Despite this substantial body of evidence, much of the existing literature has primarily focused on direct associations between SES and depression or on single mediating mechanisms. As a result, relatively limited attention has been given to integrative frameworks that simultaneously consider multiple psychosocial and structural resources. This limitation is particularly salient in the context of older adults with disabilities, where mental health outcomes are likely shaped by the complex interplay of individual, relational, and institutional factors.

To address this gap, the present study adopts the Reserve Capacity Model (RCM) as a guiding theoretical framework. The Reserve Capacity Model explains how socioeconomic disadvantage is translated into adverse health outcomes through differential exposure to stress and unequal access to psychosocial resources [23]. Reserve capacity encompasses a range of resources, including interpersonal resources such as social support and intrapsychic resources such as self-esteem and perceived control. These resources are understood to buffer the effects of stress and are closely linked to emotional well-being. Empirical studies have shown that limited reserve capacity is associated with higher levels of depressive symptoms, particularly among socioeconomically disadvantaged populations [20,24].

Within the RCM framework, intrapsychic resources refer to internal psychological capacities that support emotional regulation and coping. Among these, self-esteem—defined as an individual’s overall evaluation of self-worth [25]—has been identified as a central psychological resource. Prior research has consistently demonstrated that lower levels of self-esteem are associated with higher levels of depressive symptoms across diverse populations, including older adults and individuals with chronic illness or disability [26,27,28]. Self-esteem may function as a protective factor by enhancing individuals’ ability to cope with stress and maintain psychological resilience under adverse socioeconomic conditions.

In contrast, structural resources refer to externally provided institutional supports that facilitate access to material and social resources. Public service utilization represents a key structural resource, reflecting the extent to which individuals are able to access formal systems of care and support. Unlike informal social support, public services operate independently of personal networks and may be particularly important for individuals with limited social ties.

The application of the RCM is particularly appropriate for the present study, as older adults with disabilities are likely to experience both heightened exposure to chronic stressors (e.g., health limitations, financial strain, social exclusion) and constrained access to psychosocial and structural resources. This dual vulnerability aligns closely with the core assumptions of the RCM, making it a theoretically suitable framework for examining how socioeconomic disadvantage is associated with mental health outcomes in this population.

Building on this framework, this study conceptualizes social support as a key mediating mechanism linking socioeconomic status to depression. Lower socioeconomic status is expected to be associated with reduced access to, and quality of, social support, which in turn is associated with higher levels of depressive symptoms. Beyond this mediating pathway, this study further extends the RCM by examining how different forms of resources condition this relationship.

By integrating these dimensions, this study proposes a moderated mediation framework in which social support mediates the relationship between socioeconomic status and depression, while self-esteem and public service utilization moderate this indirect pathway. To empirically examine this framework, this study utilizes data from the supplementary survey on persons with disabilities included in the 18th wave (2023) of the Korea Welfare Panel Study (KoWePS), a nationally representative dataset that provides detailed information on socioeconomic conditions, social resources, and health outcomes.

Based on the proposed theoretical framework, the study advances the following hypotheses:

**H1.**  *Lower socioeconomic status is associated with higher levels of depression among older adults with disabilities.*

**H2.**  *Social support mediates the relationship between socioeconomic status and depression.*

**H3.**  *Self-esteem moderates the indirect effect of socioeconomic status on depression through social support.*

**H4.**  *Public service utilization moderates the indirect effect of socioeconomic status on depression through social support.*

This study contributes to the literature in three important ways. First, it extends the application of the Reserve Capacity Model to older adults with disabilities, a population that has received relatively limited attention in this theoretical framework. Second, it adopts an integrative approach by simultaneously examining psychosocial resources (social support, self-esteem) and structural resources (public service utilization). Third, it tests a moderated mediation model, thereby providing a more comprehensive understanding of how socioeconomic disadvantage is associated with depression in later life.

## 2. Materials and Methods

### 2.1. Study Design

This study employed a moderated mediation framework to examine the pathways linking socioeconomic status (SES) to depression among older adults with disabilities. The proposed model is grounded in the Reserve Capacity Model (RCM) developed by Gallo and Matthews (2003), which posits that socioeconomic disadvantage affects health outcomes through differential exposure to stress and unequal access to psychosocial resources [29]. Within this framework, individuals with lower socioeconomic status are more likely to experience chronic stressors while simultaneously having fewer psychosocial resources available to buffer these stressors, thereby increasing vulnerability to adverse mental health outcomes.

**Figure 1:**
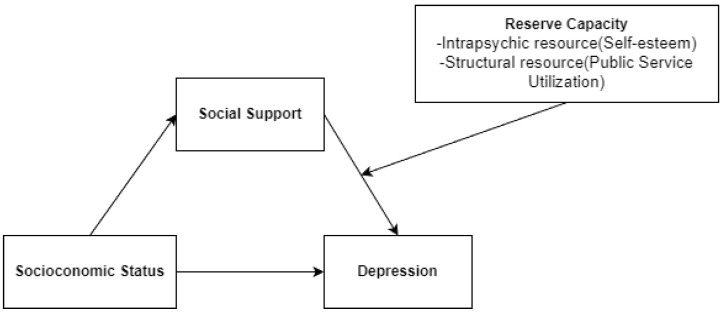
Analytic Model of the Study.

In line with this theoretical perspective, SES was hypothesized to influence depression both directly and indirectly through social support as a mediating mechanism. Specifically, lower SES was expected to be associated with reduced access to and perceived availability of social support, which in turn would increase the risk of depressive symptoms. Beyond this mediating pathway, the model further incorporates two moderating variables representing distinct dimensions of reserve capacity. Self-esteem was included as an intrapsychic resource reflecting individuals’ internal psychological capacity to cope with stress, whereas public service utilization was conceptualized as a structural resource embedded within institutional systems. These moderating factors were expected to condition the strength of the association between social support and depression, thereby shaping the indirect effect of SES on depressive symptoms. The proposed analytic model is illustrated in Figure 1.

This study was conducted and reported in accordance with the STROBE (Strengthening the Reporting of Observational Studies in Epidemiology) guidelines for observational studies.

### 2.2. Participants and Materials

This study utilized data from the supplementary survey on persons with disabilities conducted as part of the 18th wave (2023) of the KoWePS. The KoWePS is a nationally representative longitudinal dataset that provides comprehensive information on socioeconomic conditions, health status, and welfare service utilization among Korean households.

The initial sample consisted of 1576 individuals with disabilities aged 15 years and older. Participants were included if they met the following criteria: (1) aged 65 years or older, (2) identified as persons with disabilities (including both registered and non-registered individuals), and (3) provided valid responses to all key study variables (SES, social support, depression, self-esteem, and public service utilization). Cases with missing values on any of the key variables were excluded using a listwise deletion approach. After applying these criteria, the final analytic sample consisted of 845 older adults with disabilities.

The proportion of missing data across key variables was low (all <5%), minimizing the likelihood of substantial bias due to listwise deletion. Furthermore, comparisons between included and excluded cases indicated no meaningful differences in major demographic characteristics, supporting the assumption that the data were missing at random (MAR).

**Figure 2:**
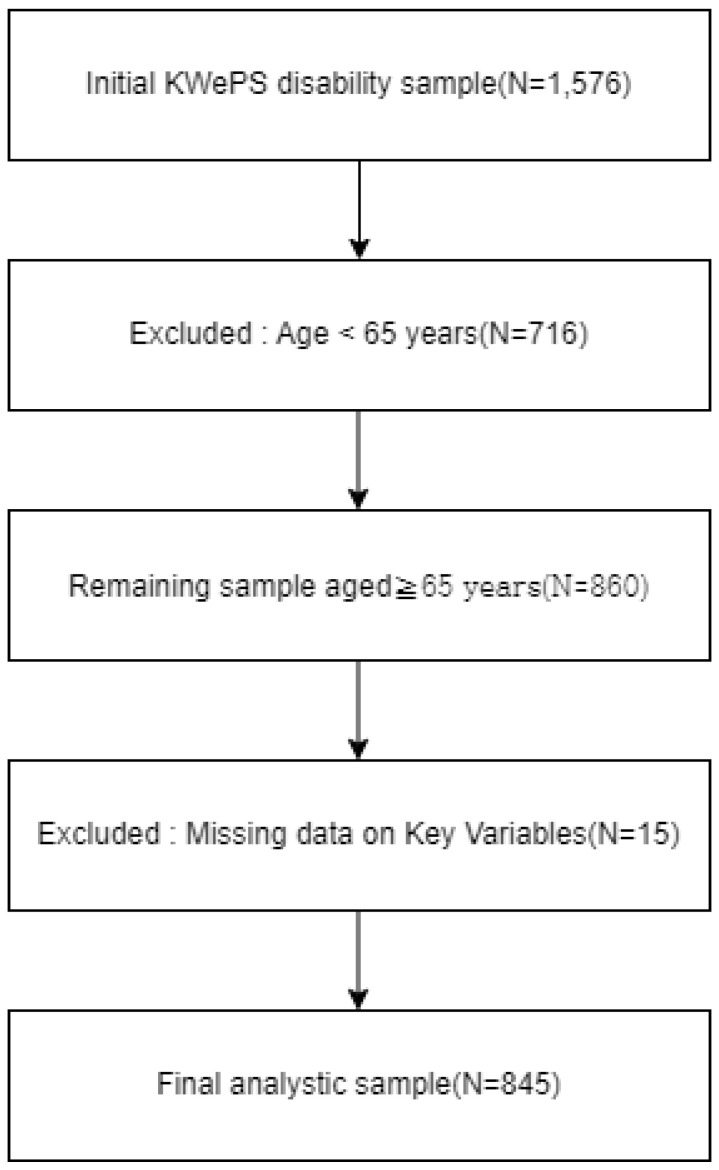
Flowchart of Sample Selection.

The sample selection process, including inclusion and exclusion criteria, is illustrated in Figure 2 to enhance transparency and reproducibility. In this study, older adults with disabilities were defined inclusively without distinguishing between individuals aging with lifelong disabilities and those experiencing late-onset disabilities. This approach was adopted to ensure sufficient statistical power and broader representativeness.

Although the KoWePS was not originally designed as a survey specifically targeting older adults with disabilities, it is a nationally representative longitudinal panel that includes a sufficiently large subsample of individuals with disabilities across the life course. The supplementary disability survey administered in the 18th wave provides detailed information on disability status, health, and service utilization, allowing for the identification and analysis of older adults with disabilities within a population-based framework. Therefore, the present study leverages the strengths of the KoWePS to examine this population while maintaining a reasonable level of representativeness at the national level.

### 2.3. Measurements of Variables

#### 2.3.1. Dependent Variable

Depression, the primary outcome variable, was measured using the 11-item version of the Center for Epidemiologic Studies Depression Scale (CES-D-11). This abbreviated scale, derived from the original 20-item CES-D developed by Radloff (1977), has been widely used in large-scale population-based studies due to its efficiency and strong psychometric properties [30]. Each item is rated on a 4-point Likert scale, with higher scores indicating greater depressive symptomatology. Positively worded items were reverse-coded prior to analysis to ensure directional consistency, and total scores were computed by summing responses across all items.

In the present study, the CES-D-11 demonstrated exceptionally high internal consistency (Cronbach’s α = 0.990), indicating strong reliability. However, such a high coefficient may reflect a high degree of inter-item correlation, potentially suggesting redundancy among items. Therefore, while the scale demonstrates excellent reliability, the findings should be interpreted with consideration of potential measurement-related limitations.

#### 2.3.2. Independent Variable

Socioeconomic status (SES), the primary independent variable, was operationalized as a composite index reflecting multiple dimensions of structural advantage. The use of composite SES indices is well supported in the literature, as single indicators may inadequately capture the multidimensional nature of socioeconomic position [28,31]. This consideration is particularly important in older populations, where income alone may not fully reflect cumulative socioeconomic conditions over the life course.

In this study, SES was constructed using three indicators: type of medical assistance, housing tenure, and educational attainment. Each indicator was dichotomized to reflect relative socioeconomic advantage and subsequently summed to produce an index ranging from 0 to 3, with higher scores indicating higher SES. Medical assistance status captured economic vulnerability, housing tenure reflected residential stability, and educational attainment represented human capital. By integrating these complementary dimensions, the composite index captures cumulative structural advantage across multiple domains and provides a more comprehensive assessment of socioeconomic inequality, consistent with prior research in aging populations.

#### 2.3.3. Mediator: Social Support

Social support was measured using a modified version of the Multidimensional Scale of Perceived Social Support (MSPSS), originally developed by Zimet et al. (1988) [32]. This instrument assesses perceived support from multiple sources, including family, friends, and significant others, and has been widely validated across diverse populations, including in the Korean context. The scale consists of 10 items rated on a 5-point Likert scale, with higher scores indicating greater perceived social support. The total score was calculated by summing all item responses. In the present study, the scale demonstrated high internal consistency (Cronbach’s α = 0.922).

#### 2.3.4. Moderators

(1) Intrapsychic resource: Self-esteem

Self-esteem was conceptualized as an intrapsychic resource within the Reserve Capacity Model and was measured using the Rosenberg Self-Esteem Scale (RSES). The RSES is one of the most widely used instruments for assessing global self-worth and has been extensively validated across diverse populations. The scale consists of 10 items rated on a 4-point Likert scale, with higher scores indicating higher levels of self-esteem. Negatively worded items were reverse-coded prior to analysis, and a total score was computed by summing all items. In the present study, internal consistency was high (Cronbach’s α = 0.899).

(2) Structural Resource: Public Service Utilization

Public service utilization was operationalized as a structural external resource reflecting access to formal institutional support. This operationalization is consistent with prior extensions of the Reserve Capacity Model, which emphasize that structural and institutional resources—including access to formal services—constitute an important component of reserve capacity, particularly among socioeconomically disadvantaged populations [33,34]. In this context, public service utilization can be understood as a behavioral indicator of access to institutional resources that may buffer the adverse effects of socioeconomic disadvantage.

This variable was constructed based on respondents’ current use of public services designed for older adults with disabilities. Five service categories were included: emotional support services, daily living support services, housing support services, household assistance services, and physical therapy and rehabilitation services. Each service was coded dichotomously (0 = not used, 1 = used), and the total score was calculated by summing across service types, yielding a range from 0 to 5.

This measure follows a formative measurement approach, in which distinct service types collectively define the construct rather than reflect a single underlying latent variable. Accordingly, internal consistency measures such as Cronbach’s alpha are not applicable.

#### 2.3.5. Control Variables

Several control variables were included in the analysis to account for potential confounding effects based on prior literature on depression among older adults with disabilities. These variables capture key demographic and life-course characteristics that may influence both social resources and mental health outcomes. Gender was included as a binary variable, coded as 0 for female and 1 for male. Marital status was recoded to distinguish between individuals with and without a spouse. Respondents who were widowed, divorced, separated, never married (including unmarried adults and single parents), or classified as other were coded as 0, while those currently married or living with a spouse were coded as 1. Age was measured as a continuous variable, calculated as the respondent’s chronological age at the time of the survey (2023), derived by subtracting the reported year of birth from the survey year. In addition, a variable distinguishing between aging with disability and late-onset disability was included to capture differences in life-course trajectories. This variable was constructed based on the reported age at disability onset. Individuals who experienced disability onset at age 49 or younger were coded as 0 (aging with disability), whereas those whose disability onset occurred at age 50 or older were coded as 1 (late-onset disability). By including these control variables, the analysis aims to more accurately isolate the relationships among socioeconomic status, social support, reserve capacity, and depression.

### 2.4. Analytic Strategy

All analyses were conducted using SPSS version 25.0 in conjunction with the PROCESS macro (version 4.2) developed by Hayes. The analytical approach was designed to systematically examine mediation, moderation, and moderated mediation effects within a unified framework.

Descriptive statistics and frequency analyses were first conducted to characterize the sample and examine the distribution of key variables. This was followed by correlation analyses and group comparisons to assess bivariate relationships and ensure that the assumptions for multivariate analyses were met.

The hypothesized mediation, moderation, and moderated mediation effects were then tested using PROCESS models. Specifically, mediation was examined using Model 4, moderation using Model 1, and moderated mediation using Model 14. This approach enabled the estimation of conditional indirect effects, allowing for the assessment of whether the mediating role of social support varies as a function of reserve capacity. To evaluate statistical significance, a bootstrapping procedure with 5000 resamples was employed to generate bias-corrected 95% confidence intervals. This non-parametric approach does not assume normality and is considered more robust than traditional methods. Effects were deemed statistically significant when the confidence interval did not include zero.

Cases with missing data were excluded using a complete-case approach. Given the low proportion of missing data and evidence supporting the assumption that data were missing at random (MAR), this approach was considered appropriate for model-based inference.

To assess the robustness of the findings, several sensitivity analyses were conducted. Alternative model specifications were tested by entering individual SES components separately rather than as a composite index, and additional models were estimated excluding public service utilization to examine the sensitivity of results to the operationalization of structural resources. Variables were also mean-centered, and interaction terms were re-estimated to confirm the stability of moderation effects. These analyses yielded substantively consistent results in both direction and statistical significance, reinforcing the robustness and reliability of the main findings.

## 3. Results

### 3.1. Demographic Information of Participants

The sociodemographic characteristics of the study participants are presented in Table 1. In terms of gender distribution, 42.6% of the sample were male and 57.4% were female, indicating a slightly higher proportion of women among older adults with disabilities. With regard to educational attainment, 64.9% of participants had completed elementary school or less, while 35.1% had attained at least a middle school education. This distribution suggests that the overall educational level of the sample is relatively low, reflecting the historical and structural constraints faced by older cohorts in accessing formal education. In terms of marital status, the largest proportion of participants were currently married (52.3%), followed by those who were widowed (38.1%). Smaller proportions were divorced (7.6%), never married (1.7%), or separated (0.4%). This pattern highlights the prevalence of marital transitions, particularly widowhood, among older adults. Regarding age distribution, nearly half of the participants (48.6%) were aged between 75 and 84 years, followed by those aged 65 to 74 years (32.0%) and those aged 85 years and older (19.4%). This indicates that the sample is largely concentrated in the mid-to-old-old age groups, which may have important implications for health status and service needs.

### 3.2. Descriptive and Correlational Statistics

The descriptive statistics for the main study variables are presented in Table 2. Socioeconomic status (SES), the independent variable, ranged from 0 to 3, with a mean of 1.493 (SD = 0.754), indicating a moderate level of socioeconomic resources within the sample. Depression, the dependent variable, ranged from 11 to 38, with a mean of 16.898 (SD = 5.609). Notably, this mean value slightly exceeds the commonly used cutoff score of 16 for identifying individuals at risk of clinically significant depressive symptoms, suggesting a relatively elevated level of depression among older adults with disabilities in this sample. Perceived social support ranged from 10 to 50, with a mean of 33.083 (SD = 7.687), indicating a moderate level of perceived support. Self-esteem scores ranged from 15 to 31, with a mean of 21.066 (SD = 2.390), reflecting a relatively stable distribution of intrapsychic resources among participants. Public service utilization ranged from 0 to 5, with a mean of 0.744 (SD = 1.049), suggesting generally low levels of engagement with formal support services. To assess the normality of the data, skewness and kurtosis values were examined for all variables. The absolute values of skewness were below 3, and kurtosis values were below 8, indicating no significant deviations from normality (West, Finch, & Curran, 1995) [35]. These results support the appropriateness of subsequent parametric analyses.

Prior to testing the hypothesized model, Pearson correlation analyses were conducted to examine the relationships among the study variables. The results are presented in Table 3. Socioeconomic status (SES), the independent variable, was negatively correlated with depression (r = −0.204, *p* < 0.01) and public service utilization (r = −0.374, *p* < 0.01), while showing a positive correlation with social support (r = 0.269, *p* < 0.01). These findings suggest that individuals with higher socioeconomic status tend to experience lower levels of depression while having greater access to social support. Depression, the dependent variable, was negatively associated with social support (r = −0.193, *p* < 0.01), self-esteem (r = −0.192, *p* < 0.01), and positively associated with public service utilization (r = 0.307, *p* < 0.01). The mediating variable, social support, was positively correlated with self-esteem (r = 0.159, *p* < 0.01) and negatively correlated with public service utilization (r = −0.171, *p* < 0.01). This suggests that individuals with stronger perceived social support tend to possess greater intrapsychic resources and well-being, while relying less on formal support systems. To assess the potential issue of multicollinearity, both correlation coefficients and variance inflation factors (VIF) were examined. All correlation coefficients were below 0.40 in absolute value, indicating low to moderate associations among variables. Additionally, all VIF values were below the commonly accepted threshold of 10, confirming that multicollinearity was not a concern in the present analysis.

### 3.3. Mediating Effect of Social Support in the Relationship Between SES and Depression

To examine the mediating role of social support in the relationship between SES and depression, a mediation analysis was conducted using the PROCESS macro. The results are presented in Table 4. First, the total effect of SES on depression was found to be statistically significant and negative (B = −0.978, *p* < 0.001), indicating that higher levels of socioeconomic status are associated with lower levels of depression among older adults with disabilities. Second, SES was positively associated with social support (B = 2.012, *p* < 0.001), suggesting that individuals with higher socioeconomic status tend to report greater perceived social support. In turn, social support was significantly and negatively associated with depression (B = −0.110, *p* < 0.001), indicating that higher levels of social support are linked to lower levels of depressive symptoms. When the mediating variable was included in the model, the direct effect of SES on depression (c′) remained statistically significant (B = −0.756), but its magnitude was reduced compared to the total effect (B = −0.978). This reduction in effect size indicates that social support partially mediates the relationship between SES and depression. Taken together, these findings provide empirical support for the mediating role of social support, suggesting that socioeconomic status influences depression both directly and indirectly through its impact on perceived social support.

To further assess the statistical significance of the mediating effect, a bootstrapping procedure was conducted, and the results are presented in Table 5. The estimated indirect effect of socioeconomic status (SES) on depression through social support was −0.222. The bootstrapped confidence interval for the indirect effect ranged from −0.383 to −0.094. Because this interval does not include zero, the indirect effect is considered statistically significant. This finding provides robust evidence for the mediating role of social support. In other words, socioeconomic status not only exerts a direct effect on depression but also influences depression indirectly through its impact on perceived social support. The persistence of a significant direct effect alongside a significant indirect effect indicates that social support functions as a partial mediator in the relationship between SES and depression among older adults with disabilities.

### 3.4. Moderation Effects of Reserve Capacity

#### 3.4.1. Intrapsychic Resource: Self-Esteem

To examine whether self-esteem moderates the relationship between social support and depression among older adults with disabilities, a moderation analysis was conducted. The results are presented in Table 6. In the final step, the inclusion of the interaction term led to a significant increase in explained variance (ΔR^2^ = 0.0044, *p* < 0.05), indicating a statistically significant moderating effect of self-esteem. Specifically, the negative association between social support and depression was stronger among individuals with higher levels of self-esteem compared to those with lower levels. Further analysis of the conditional effects revealed that the impact of social support on depression was statistically significant at both the mean level of self-esteem (M) and at one standard deviation above the mean (M + 1 SD).

#### 3.4.2. Structural Resource: Service Utilization

To examine whether service utilization moderates the relationship between social support and depression among older adults with disabilities, a moderation analysis was conducted. The results are presented in Table 7. In the final step, the inclusion of the interaction term resulted in a significant increase in explained variance (ΔR^2^ = 0.0068, *p* < 0.001), indicating a statistically significant moderating effect of service utilization. Specifically, the protective effect of social support on depression weakened as the level of service utilization increased. Further analysis of the conditional effects revealed that the effect of social support on depression was statistically significant at the mean level of service utilization (M) and at one standard deviation below the mean (M − 1 SD).

### 3.5. Moderated Mediating Effects

#### 3.5.1. Intrapsychic Resource (Self-Esteem)

The moderated mediation effect of self-esteem, conceptualized as an intrapsychic resource, was examined to assess whether it conditions the indirect effect of socioeconomic status (SES) on depression through social support. The detailed results are presented in Table 8. First, the model predicting social support was statistically significant (R^2^ = 0.091, *F* = 16.827, *p* < 0.001). SES exhibited a significant positive effect on social support (B = 2.223, SE = 0.363, *t* = 6.131, *p* < 0.001), indicating that individuals with higher SES reported greater levels of perceived social support. Among the control variables, marital status was also significant (B = 2.348, *p* < 0.001), suggesting that socially embedded relationships may play a role in shaping perceived support.

Next, the model predicting depression was statistically significant (R^2^ = 0.141, *F* = 17.174, *p* < 0.001). SES showed a significant negative association with depression (B = −0.867, SE = 0.264, *t* = −3.287, *p* < 0.01), indicating that higher SES is associated with lower levels of depressive symptoms. However, neither social support (B = 0.301, SE = 0.203, *t* = 1.486, n.s.) nor self-esteem (B = 0.271, SE = 0.322, *t* = 0.841, n.s.) demonstrated statistically significant main effects. The interaction term between social support and self-esteem (A × B) was negative (B = −0.018, SE = 0.009, *t* = −1.943) and approached statistical significance but did not reach conventional levels of significance. This indicates that the moderating role of self-esteem in the relationship between social support and depression was not supported. Among the control variables, gender (B = −1.286, *p* < 0.01) and marital status (B = −1.160, *p* < 0.01) were significant predictors of depression, indicating that depressive symptoms vary across demographic and relational contexts, whereas age was not significant.

Taken together, these findings suggest that self-esteem does not significantly moderate the mediating pathway linking SES, social support, and depression. In other words, the conditional indirect effect of SES on depression through social support, as a function of self-esteem, was not supported.

#### 3.5.2. Structural Resource (Service Utilization)

To examine whether public service utilization moderates the indirect effect of socioeconomic status (SES) on depression through social support, a moderated mediation analysis was conducted. The results are presented in Table 9. First, the model predicting social support as the dependent variable was statistically significant (*F* = 17.002, *p* < 0.001), explaining approximately 9% of the variance. Among the control variables, marital status was found to be a significant predictor (B = 2.348, *p* < 0.001). In addition, SES exhibited a significant positive main effect on social support (B = 2.223, *p* < 0.001), indicating that individuals with higher SES reported greater levels of perceived social support.

Next, the model predicting depression was also statistically significant (*F* = 21.083, *p* < 0.001), accounting for 16.8% of the variance. Within this model, social support was negatively associated with depression (B = −0.139, *p* < 0.001), suggesting that higher levels of social support are linked to lower levels of depressive symptoms. Importantly, the interaction term between social support and public service utilization was statistically significant and positively associated with depression (B = 0.057, *p* < 0.01). This indicates that the strength of the association between social support and depression varies depending on the level of public service utilization. Among the control variables, gender was also significant (B = −1.047, *p* < 0.05), indicating that females were more vulnerable to depressive symptoms. Taken together, these findings suggest that public service utilization functions as a significant moderator in the relationship between social support and depression, thereby supporting the presence of a moderated mediation mechanism in which the indirect effect of SES on depression via social support varies across levels of structural resource availability.

To assess the statistical significance of the moderated mediation effect, the conditional indirect effects of socioeconomic status (SES) on depression through social support were estimated at different levels of public service utilization using a bootstrapping procedure. The results are presented in Table 10. The index of moderated mediation was estimated at 0.113, and its 95% confidence interval ranged from 0.010 to 0.238. As the confidence interval did not include zero, the moderated mediation effect was statistically significant. These findings provide robust evidence that the indirect effect of SES on depression via social support is contingent upon the level of public service utilization, thereby supporting the presence of a conditional indirect effect within the proposed analytical framework.

The results of the conditional indirect effect analysis are presented in Table 11. The findings indicate that the magnitude of the indirect effect of socioeconomic status (SES) on depression through social support varies systematically across levels of public service utilization. Specifically, at lower (−1 SD) and mean levels of service utilization, the conditional indirect effects were statistically significant, as their 95% bootstrapped confidence intervals did not include zero (−1 SD: B = −0.282, 95%CI [−0.471, −0.130]; Mean: B = −0.198, 95%CI [−0.352, −0.074]). These results suggest that lower SES is associated with higher levels of depression through reduced social support among individuals with low to moderate levels of public service utilization. In contrast, at higher levels of service utilization (+1 SD), the conditional indirect effect was not statistically significant (B = −0.079, 95%CI [−0.255, 0.080]), as the confidence interval included zero. This indicates that the indirect pathway linking SES to depression via social support is attenuated and no longer significant among individuals with higher levels of public service utilization.

Taken together, these findings demonstrate that the indirect effect of SES on depression through social support diminishes as the level of public service utilization increases, providing further evidence of a significant moderated mediation effect. In substantive terms, the detrimental pathway from low SES to increased depression via reduced social support appears to operate primarily among individuals with limited access to public services, whereas this pathway is effectively neutralized among those with higher levels of service utilization.

## 4. Discussion

This study examined the relationships among socioeconomic status (SES), social support, reserve capacity, and depression among older adults with disabilities within the framework of the Reserve Capacity Model (RCM). By integrating both mediating and conditional pathways, the findings provide a structured account of how socioeconomic disadvantage is differentially associated with mental health outcomes in this population.

The findings indicate that SES is associated with depression both directly and indirectly through social support. Specifically, lower SES was associated with lower levels of perceived social support, and this pattern was in turn associated with higher levels of depressive symptoms. These results are broadly consistent with prior literature documenting socioeconomic gradients in mental health and the role of social relationships in later life. However, given the cross-sectional design, these associations should be interpreted as observed patterns rather than as evidence of directional or causal pathways.

A notable aspect of the findings is that the direct association between social support and depression was not statistically significant in the full model, despite the presence of a statistically significant indirect effect. This pattern suggests that the mediating role of social support should be interpreted as reflecting the joint distribution of relationships among SES, social support, and depression, rather than as evidence of a strong independent association between social support and depressive symptoms. Similar patterns have been reported in mediation analyses where indirect effects emerge despite non-significant direct paths, particularly when multiple pathways operate simultaneously or when suppression or shared variance is present. Accordingly, the present findings indicate that social support remains relevant within the broader relational structure linking SES and depression, although its independent contribution appears limited within the fully adjusted model.

With respect to reserve capacity, public service utilization demonstrated a statistically significant moderating effect, whereas self-esteem did not. The absence of a significant moderating role for self-esteem indicates that, within the present data, there is no empirical support for the hypothesis that intrapsychic resources condition the association between social support and depression. This result should be interpreted as a null finding rather than as evidence against the theoretical relevance of self-esteem. It is possible that the role of intrapsychic resources may depend on factors not captured in the current analysis, such as severity of disability, cumulative stress exposure, or contextual variations in social environments.

In contrast, the moderating role of public service utilization suggests that the association between social support and depression varies depending on the level of engagement with formal support systems. The conditional effects indicate that the association between social support and depression was more pronounced at lower and moderate levels of public service utilization and attenuated at higher levels. Although no formal tests of nonlinearity (e.g., quadratic terms or Johnson–Neyman intervals) were conducted, this pattern is consistent with a potential threshold-like dynamic in which formal services may partially compensate for limited informal support under certain conditions.

Importantly, this pattern should not be interpreted as evidence that public service utilization reduces depression or replaces social support. An alternative and equally plausible interpretation is that individuals with higher needs—such as those experiencing greater social isolation or health limitations—are more likely to utilize public services. Thus, public service utilization may reflect underlying need and service engagement rather than exerting an independent protective effect. The observed moderation therefore highlights heterogeneity in how social and structural resources are jointly associated with depression, rather than indicating a unidirectional or causal buffering mechanism.

Despite these interpretive constraints, the findings provide several targeted implications for policy and service system design. First, the observed pattern that the association between social support and depression is strongest at lower levels of public service utilization suggests that individuals with limited access to both informal and formal resources may be particularly vulnerable. This underscores the importance of early identification strategies aimed at older adults with disabilities who are simultaneously socially isolated and underconnected to formal services.

Second, the attenuation of the association at higher levels of service utilization suggests that formal services may function as partial substitutes or complements to informal support under certain conditions. From a policy perspective, this highlights the need to strengthen the accessibility, continuity, and intensity of public service provision, particularly for socioeconomically disadvantaged individuals. For example, integrated case management systems that proactively connect individuals to multiple services may help reduce fragmentation and ensure sustained engagement with support systems.

Third, the findings suggest that service delivery systems should be designed to operate not only as providers of discrete interventions but also as coordinative platforms linking health care, social care, and community-based services. In practice, this may involve developing integrated care models, strengthening referral pathways across service sectors, and enhancing continuity through longitudinal case monitoring. Such approaches are particularly relevant for older adults with disabilities, who often navigate complex and fragmented service environments.

Importantly, these policy implications should be interpreted as being grounded in observed associations rather than as direct tests of service system effectiveness. The present study does not directly measure system-level characteristics such as service quality, coordination, or integration. Therefore, while the findings highlight the potential relevance of formal service engagement, further research is needed to identify which specific service features are most effective in improving mental health outcomes.

From a research perspective, the findings highlight several directions for future investigation. Longitudinal studies are needed to clarify temporal ordering and to assess whether changes in social support and service utilization are associated with subsequent changes in depression. In addition, analytical approaches such as structural equation modeling (SEM) could provide a more comprehensive assessment of the theoretical framework by accounting for measurement error and allowing for the estimation of latent constructs. Further research examining nonlinear effects and interaction thresholds using formal statistical techniques would also help to refine the interpretation of the moderating role of structural resources.

Several limitations should be acknowledged. First, the cross-sectional design precludes conclusions regarding temporal ordering or causality. Reverse associations are plausible; for example, depressive symptoms may influence perceptions of social support or patterns of service utilization. Second, the operationalization of key variables, including SES and public service utilization, relies on composite indices that may not fully capture their multidimensional nature. Third, the findings are based on data from a single national context, and their generalizability to other institutional settings may be limited.

In conclusion, the present study contributes to the literature by applying the Reserve Capacity Model to older adults with disabilities and by jointly examining psychosocial and structural dimensions of resources. The findings suggest that socioeconomic disadvantage, social support, and engagement with formal services are interrelated in their associations with depression. Rather than indicating the primacy of one type of resource over another, the results highlight the importance of considering how multiple forms of resources operate together within specific social and institutional contexts.

## 5. Conclusions

This study provides an integrated examination of the associations between socioeconomic status (SES) and depression among older adults with disabilities within the framework of the Reserve Capacity Model. The findings indicate that SES is associated with depressive symptoms both directly and indirectly through social support, suggesting that differences in social resources are linked to socioeconomic disparities in mental health within this population. In addition, the results highlight that both psychosocial and structural dimensions of resources are relevant for understanding these associations.

The findings further suggest that the association between social support and depression varies according to levels of engagement with formal support systems. While this pattern points to the potential relevance of public service utilization in shaping observed relationships, it should be interpreted as reflecting conditional associations rather than as evidence of causal or threshold effects, given the cross-sectional design and the absence of formal nonlinearity testing.

From a policy perspective, the results underscore the importance of considering both informal and formal support systems when addressing mental health disparities among older adults with disabilities. In particular, efforts to improve accessibility to public services and to reduce barriers to utilization may be especially relevant for individuals with limited social support. Additionally, strengthening continuity of care and enhancing linkages across health, social care, and community-based services may contribute to more coordinated and responsive service delivery. However, as the present study does not directly assess service system characteristics, these implications should be interpreted as empirically informed directions rather than definitive policy conclusions.

From a practice perspective, the findings suggest that interventions targeting depression in this population may benefit from approaches that integrate psychosocial support with facilitation of access to formal services. This highlights the importance of addressing both individual-level and structural contexts in clinical and community-based interventions.

Overall, this study contributes to the literature by applying the Reserve Capacity Model to older adults with disabilities and by jointly examining psychosocial and structural resources in relation to mental health outcomes. Future research using longitudinal or experimental designs is needed to clarify temporal ordering and to further investigate the mechanisms underlying these associations. In addition, greater attention to the specific features of service systems, including accessibility, continuity, and coordination, may help to advance understanding of how institutional contexts are linked to mental health outcomes in this population.

## Figures and Tables

**Table 1:** Demographic information of the participants (N = 845).

Characteristics	Categories	N (%)
Gender	Male	360 (42.6)
	Female	485 (57.4)
Age	65~74	270 (32.0)
	75~84	411 (48.6)
	85 and over	164 (19.4)
Education	No formal Education	150 (17.8)
	Elementary school	398 (471)
	Middle school	156 (18.5)
	High school	113 (13.4)
	College Graduate and over	28 (3.3)
Marital Status	Married	442 (52.3)
	Widowed(er)	322 (38.1)
	Divorced	64 (7.6)
	Separated	3 (0.4)
	Never been married	14 (1.7)

**Table 2:** Descriptive statistics of variables (N = 845).

Variables		Min	Max	Mean	SD	Skewness	Kutosis
SES		0.00	3.00	1.4935	0.75352	−0.761	−0.362
Depression		11.000	38.000	16.8982	5.609	1.182	0.989
Social Support		10.00	50.00	33.0828	7.6868	−0.465	0.099
Reserve Capacity	Self-esteem	15.00	31.00	21.0663	2.3903	0.398	0.640
	Service Utilization	0.00	5.00	0.7444	1.0490	1.432	1.447

**Table 3:** Correlation analysis of the variables (N = 845).

Variables		1	2	3	4	5
1. SES		1				
2. Depression		−0.204 **	1			
3. Social Support		0.269 **	−0.193 **	1		
Reserve Capacity	4. Self-esteem	0.056	−0.192 **	0.159 **	1	
	5. Service Utilization	−0.374 **	0.307 **	−0.171 **	−0.059	1

** *p* < 0.01.

**Table 4:** The Mediation Analysis (N = 845).

Dependent Variables	Independent Variables		Β	SE	T	F	R-sq
Depression	SES		−0.978	0.242	−4.049 ***	17.745 ***	0.096
	Control variables	Sex	−1.241	0.418	−2.969 **		
		Marriage status	−1.241	0.434	−2.856 **		
		Age	0.077	0.029	2.628 **		
		Late-onset disability	0.287	0.449	0.638		
Social Support	SES		2.012	0.332	6.063 ***	16.827 ***	0.091
	Control variables	Sex	−1.309	0.574	−2.279 *		
		Marriage status	2.291	0.597	3.838 ***		
		Age	0.061	0.041	1.504		
		Late-onset disability	0.442	0.617	0.716		
Depression	SES		−0.756	0.244	−3.099 **	0.002	0.116
	Social Support		−0.11	0.025	−4.435 ***		
	Control variables	Sex	−1.385	0.415	−3.341 **		
		Marriage status	−0.988	0.433	−2.28 *		
		Age	0.084	0.029	2.883 **		
		Late-onset disability	0.336	0.444	0.755		

* *p* < 0.05, ** *p* < 0.01, *** *p* < 0.01.

**Table 5:** Bootstrapping Results for the Mediating Effect of Social Support.

Variables	Index of Mediation	BootSE	95%CI	
			LLCI	ULCI
Social Support	−0.222	0.073	−0.383	−0.094

**Table 6:** Moderation effects of intrapsychic resource (Self-esteem).

Dependent Variables	Independent Variables		Β	SE	T		95%CI	
							LLCI	ULCI
Depression	Social Support (A)		−0.1029	0.0246	−4.1831 ***		−0.1512	−0.0546
	Self-esteem (B)		−0.3486	0.0778	−4.4779 ***		−0.5014	−0.1958
	A × B		−0.0196	0.0095	−2.0559 *		−0.0383	−0.0009
	Control variables	Sex	−1.2349	0.4129	−2.991 **		−2.0452	−0.4245
		Marriage status	−1.6032	0.4074	−3.9352 ***		−2.4029	−0.8035
		Age	0.0772	0.029	2.6624 **		0.0203	0.1342
		Late-onset disability	0.1902	0.4388	0.4333		−0.6712	1.0515
**Change in R^2^ Due to Interaction**			**R^2^**			**F**		
			0.0044			4.2266 *		
**Conditional Effect**			**B**	**SE**			**T**	
−1 SD			−0.0561	0.0346			−1.6212	
M			−0.1029	0.0246			−4.1831 ***	
+1 SD			−0.1497	0.0324			−4.6176 ***	

* *p* < 0.05, ** *p* < 0.01, *** *p* < 0.01.

**Table 7:** Moderation effects of Structural resource (Service utilization).

Dependent Variables	Independent Variables		Β	SE	T		95%CI	
							LLCI	ULCI
Depression	Social Support (A)		−0.1015	0.024	−4.2296 ***		−0.1487	−0.0544
	Service utilization (B)		1.4362	0.187	7.6781 ***		1.069	1.8033
	A × B		0.0567	0.0218	2.608 **		0.014	0.0994
	Control variables	Sex	−1.0048	0.4065	−2.4719 *		−1.8026	−0.2069
		Marriage status	−0.873	0.4041	−2.1602 *		−1.6663	−0.0798
		Age	0.054	0.0287	1.8835		−0.0023	0.1103
		Late-onset disability	0.3892	0.4308	0.9035		−0.4564	1.2348
**Change in R^2^ Due to Interaction**			**R^2^**			**F**		
			0.0068			6.8017 **		
**Conditional Effect**			**B**	**SE**			**T**	
−1 SD			−0.1438	0.0297			−4.8339 ***	
M			−0.1015	0.024			−4.2296 ***	
+1 SD			−0.042	0.0321			−1.3084	

* *p* < 0.05, ** *p* < 0.01, *** *p* < 0.01.

**Table 8:** Moderated Mediating Effects: Intrapsychic Resource (Self-esteem).

Dependent Variable	Independent Variable		Coeff	SE	T	LLCI	ULCI	R^2^	F
Social Support	SES		2.223	0.363	6.131 ***	1.511	2.935	0.091 ***	16.827
	Control variables	Sex	−1.111	0.574	−1.935	−2.239	0.016		
		Marriage status	2.348	0.592	3.963 ***	1.185	3.511		
		Age	0.451	0.617	0.731	−0.76	1.661		
		Late-onset disability	0.033	0.04	0.829	−0.046	0.112		
Depression	SES		−0.867	0.264	−3.287 **	−1.384	−0.349	0.141 ***	17.174
	Social Support (A)		0.301	0.203	1.486	−0.097	0.699		
	Self-esteem (B)		0.271	0.322	0.841	−0.361	0.903		
	A × B		−0.018	0.009	−1.943	−0.037	0		
	Control variables	Sex	−1.286	0.411	−3.131 **	−2.092	−0.48		
		Marriage status	−1.16	0.427	−2.716 **	−1.998	−0.322		
		Age	0.326	0.438	0.744	−0.534	1.186		
		Late-onset disability	0.077	0.029	2.669 **	0.02	0.134		

** *p* < 0.01, *** *p* < 0.01.

**Table 9:** Moderated Mediating Effects: Structural Resource (Service utilization).

Dependent Variable	Independent Variable		Coeff	SE	T	LLCI	ULCI	R^2^	F
Social Support	SES		2.223	0.363	6.131 ***	1.511	2.935	0.092 ***	17.002
	Control variables	Sex	−1.111	0.574	−1.935	−2.239	0.016		
		Marriage status	2.348	0.592	3.963 ***	1.185	3.511		
		Age	0.451	0.617	0.731	−0.76	1.661		
		Late-onset Disability	0.033	0.04	0.829	−0.046	0.112		
Depression	SES		−0.391	0.273	−1.432	−0.927	0.145	0.168 ***	21.083
	Social Support (A)		−0.139	0.03	−4.625 ***	−0.197	−0.08		
	Service utilization (B)		−0.544	0.705	−0.772	−1.928	0.84		
	A × B		0.057	0.022	2.635 **	0.015	0.1		
	Control variables	Sex	−1.047	0.407	−2.57 *	−1.846	−0.247		
		Marriage status	−0.707	0.42	−1.684	−1.532	0.117		
		Age	0.438	0.432	1.014	−0.41	1.286		
		Late-onset Disability	0.056	0.029	1.954	0	0.112		

* *p* < 0.05, ** *p* < 0.01, *** *p* < 0.01.

**Table 10:** Index of Moderated Mediation for Public Service Utilization.

Index of Moderated Mediation	BootSE	95%CL	
		LLCI	ULCI
0.113	0.057	0.01	0.238

**Table 11:** The Moderated Mediating Effects According to the Values of Service Utilization.

Service Utilization	Conditional Indirect Effects at Specific Values of the Moderator			
	Effect	BootSE	LLCI	ULCI
−1 SD	−0.282	0.087	−0.471	−0.13
M	−0.198	0.07	−0.352	−0.074
+1 SD	−0.079	0.085	−0.255	0.08

## Data Availability

The original data presented in the study are openly available at https://www.koweps.re.kr:442/main.do;jssionid=2912AA7A075BE8A7FE6772AD75BDB6A6 (accessed on 10 February 2025).
